# Congenital Myasthenic Syndrome Type 19 Is Caused by Mutations in *COL13A1*, Encoding the Atypical Non-fibrillar Collagen Type XIII α1 Chain

**DOI:** 10.1016/j.ajhg.2015.10.017

**Published:** 2015-11-25

**Authors:** Clare V. Logan, Judith Cossins, Pedro M. Rodríguez Cruz, David A. Parry, Susan Maxwell, Pilar Martínez-Martínez, Joey Riepsaame, Zakia A. Abdelhamed, Alice V.R. Lake, Maria Moran, Stephanie Robb, Gabriel Chow, Caroline Sewry, Philip M. Hopkins, Eamonn Sheridan, Sandeep Jayawant, Jacqueline Palace, Colin A. Johnson, David Beeson

**Affiliations:** 1 Section of Ophthalmology & Neurosciences, Leeds Institute of Biomedical and Clinical Sciences, University of Leeds, Leeds LS9 7TF, UK; 2 Neurosciences Group, Nuffield Department of Clinical Neurosciences, Weatherall Institute of Molecular Medicine, University of Oxford, Oxford OX3 9DS, UK; 3 Section of Genetics, Leeds Institute of Biomedical and Clinical Sciences, University of Leeds, Leeds LS9 7TF, UK; 4 Neuroimmunology Group, Division of Neuroscience, School for Mental Health and Neuroscience, Maastricht University, PO box 616, 6200 MD Maastricht, the Netherlands; 5 MRC Molecular Haematology Unit, Weatherall Institute of Molecular Medicine, University of Oxford, Oxford OX3 9DS, UK; 6 Department of Paediatric Neurology, Nottingham City Hospital, Nottingham University Hospitals NHS Trust, Hucknall Road, Nottingham NG5 1PB, UK; 7 Dubowitz Neuromuscular Centre, Institute of Child Health and Great Ormond Street Hospital, 30 Guilford Street, London WC1N 1EH, UK; 8 Section of Translational Anaesthesia and Surgical Sciences, Leeds Institute of Biomedical and Clinical Sciences, University of Leeds, Leeds LS9 7TF, UK; 9 Department of Paediatric Neurology, John Radcliffe Hospital, Oxford Radcliffe Hospitals NHS Trust, Oxford OX3 9DU, UK; 10 Department of Clinical Neurology, John Radcliffe Hospital, Oxford Radcliffe Hospitals NHS Trust, Oxford OX3 9DU, UK

## Abstract

The neuromuscular junction (NMJ) consists of a tripartite synapse with a presynaptic nerve terminal, Schwann cells that ensheathe the terminal bouton, and a highly specialized postsynaptic membrane. Synaptic structural integrity is crucial for efficient signal transmission. Congenital myasthenic syndromes (CMSs) are a heterogeneous group of inherited disorders that result from impaired neuromuscular transmission, caused by mutations in genes encoding proteins that are involved in synaptic transmission and in forming and maintaining the structural integrity of NMJs. To identify further causes of CMSs, we performed whole-exome sequencing (WES) in families without an identified mutation in known CMS-associated genes. In two families affected by a previously undefined CMS, we identified homozygous loss-of-function mutations in *COL13A1*, which encodes the alpha chain of an atypical non-fibrillar collagen with a single transmembrane domain. COL13A1 localized to the human muscle motor endplate. Using CRISPR-Cas9 genome editing, modeling of the *COL13A1* c.1171delG (p.Leu392Sfs^∗^71) frameshift mutation in the C2C12 cell line reduced acetylcholine receptor (AChR) clustering during myotube differentiation. This highlights the crucial role of collagen XIII in the formation and maintenance of the NMJ. Our results therefore delineate a myasthenic disorder that is caused by loss-of-function mutations in *COL13A1*, encoding a protein involved in organization of the NMJ, and emphasize the importance of appropriate symptomatic treatment for these individuals.

## Main Text

The neuromuscular junction (NMJ) is a specialized synapse formed and maintained through interaction of three main structural components: the motor nerve terminal, postsynaptic muscle membrane, and synapse-associated terminal Schwann cells.^1^ Congenital myasthenic syndromes (CMSs) are inherited disorders of signal transmission at the NMJ, and they demonstrate considerable clinical variability and genetic heterogeneity.^2^ All CMS types share the clinical feature of fatigable weakness, but age of onset, manifesting symptoms, distribution of weakness, disease progression, and response to treatment differ depending on the disrupted molecular mechanism resulting from genetic mutations.^3^ Accurate differential diagnosis between the various CMS sub-types and other congenital neuromuscular conditions remains an important clinical need given that CMSs respond symptomatically to appropriate treatment.^2^, ^3^

CMSs are classified according to the mutated gene and the resulting pathogenic mechanism, but in general, they can affect presynaptic, synaptic, or postsynaptic functions.^2^ Alterations in proteins involved in postsynaptic functions are the most common in that they account for 88% of CMS-affected individuals in the UK.^4^ They most notably occur in RAPSN (CMS type 11 [MIM: 616326 ]), DOK7 (CMS type 10 [MIM: 254300]), and components of the acetylcholine receptor (AChR; CMS types 1–4 [see MIM: 601462 for further details]). Mutations in, for example, *COLQ* (MIM: 603033) or *CHAT* (MIM: 118490)—causing synaptic CMS (CMS type 5 [MIM: 603034]) or presynaptic CMS (CMS type 6 [MIM: 254210]), respectively—are less frequent in that each accounts for fewer than 10% or 5% of individuals, respectively,^4^, ^5^ although this might vary in different ethnic groups. About 10% of UK individuals with a CMS have no identified mutation in the known CMS-associated genes.^4^ At present, mutations in at least 18 different genes are known to cause CMSs,^1^ but a detailed understanding of the mechanisms of NMJ formation and maturation remains incomplete. Here, we used whole-exome sequencing (WES) to identify *COL13A1* (MIM: 120350) mutations that cause a CMS form that we designate CMS type 19.

**Figure 1:**
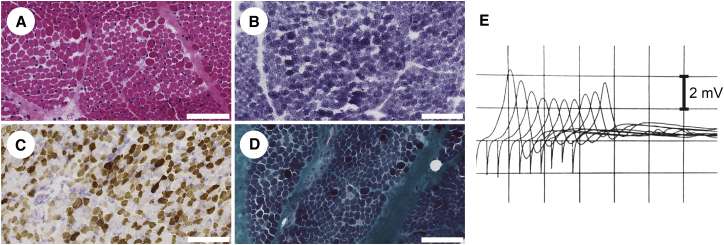
Features of Individuals with *COL13A1* Mutations (A–D) Muscle biopsy from the quadriceps muscle of affected individual II:1 from family 1 at 6 months of age. (A) H&E staining shows abnormal variation in fiber size, few central nuclei, and occasional vacant peripheral vacuole-like areas. (B) Staining for NADH-tetrazolium reductase shows occasional fibers with a halo-like appearance. (C) ATPase staining at pH 4.6 shows several populations of fibers staining positive for fetal myosin. (D) Gömöri trichrome staining shows several hypercontracted fibers and enlarged mitochondria. Fiber size ranges from 4 to 18 μm. Scale bars represent 100 μm. (E) Repetitive nerve stimulation of left flexor hallucis brevis muscle (II:1 family 1) shows significant (>20%) decrement.

Individual 1 (affected individual II:1 in family 1; Table 1) is of white European origin. She developed recurrent apneas and a poor suck soon after birth, and by 7 months, generalized hypotonia and gastresophageal reflux were noted. Examination showed mild bilateral non-fatigable ptosis with normal eye movements and no facial weakness but also showed poor head control, marked neck weakness, and limb hypotonia. There were no joint contractures. When she was 2 years old, dysmorphic features including low-set ears, micrognathia, retrognathia, a high-arched palate, and pectus carinatum (barrel chest) were noted. A muscle biopsy from the quadriceps muscle revealed abnormal variation in fiber size, and several populations of fibers stained positive for fetal myosin (Figures 1A–1D). Respiratory enzymes were normal (Figures 1A–1D), and whole-muscle MRI was normal. Levels of serum creatine kinase (CK) were within the normal range. Stimulated single-fiber electromyography (SFEMG) for the left orbicularis oculi muscle showed grossly increased jitter (mean consecutive difference [MCD] = 133.36 ± 41.9 μs) and increased blocking (14%), consistent with a diagnosis of CMS. Low rates of repetitive nerve stimulation in the right abductor digiti minimi and left flexor hallucis brevis showed significant (>20%) decrement at rest (Figure 1E). There was no response to anticholinesterase medication. Treatment with 3,4-diaminopyridine (3,4-DAP; 0.3 mg/kg/day) and salbutamol (0.56 mg/kg/day) produced a remarkable improvement in her motor and respiratory function.

Individuals 2 and 3 (affected individuals II:1 and II:2, respectively, in family 2; Table 1) are siblings with parental consanguinity and an ethnic origin from the Indian subcontinent. Individual 2 had feeding difficulties in the first year of life and was noted to have ptosis. During childhood, he had dyspnoea on exertion, recurrent chest infections, and mild learning difficulties. Neurophysiology was performed when he was 18 years old. Repetitive nerve stimulation at 3 Hz in the right anconeous muscle showed a significant (>20%) decrement. SFEMG in the right extensor digitorum communis showed 50% increased jitter (mean MCD = 69.8 ± 34.4 μs). When he was 25 years old, examination showed constant moderate bilateral ptosis (30%), normal eye movements, mild weakness of eye closure and facial muscles, and mild weakness of hip extensors. He showed facial dysmorphism including micrognatia, low-set ears, and a high-arched palate (Table 1), in addition to skeletal abnormalities such as pectus carinatum and marked bilateral pes cavus. There was no response to treatment with pyridostigmine.

Individual 3 was more severely affected than her brother. She had severe breathing and feeding difficulties after birth, partly as a result of a combined hiatus and diaphragmatic hernia. A muscle biopsy from the quadriceps muscle at age 1 year was within normal histological limits. She depended on oxygen until the age of 2 years and had recurrent chest infections, after which she developed chronic lung disease. She had delayed motor milestones. She was predominantly fed with a gastrostomy tube and had limited tolerance to walking and exercise, which caused progressive fatigue. Examination at age 5 years showed bilateral ptosis, mild limitation of eye movements, and mild facial weakness. Muscle bulk was reduced, and there was generalized mild muscle weakness. Facial dysmorphism was similar to that of her brother, individual 2. In addition, pectus carinatum and a degree of spinal rigidity with kyphotic posture, predominantly at the cervical-thoracic region, were present, but there were no distal contractures. This individual died at 8 years of age as a result of severe respiratory problems related to muscle weakness and her chronic lung disease. Additional clinical information is presented in the Supplemental Note.

Ethical approval for molecular genetics research studies and use of data was obtained from the South Yorkshire Research Ethics Committee (reference no. 11/H1310/1) and Oxford Research Ethics Committees OXREC B (04/OXB/017) and OXREC C (09/H0606/74). We obtained informed consent from all participating families or individuals. Genomic DNA was extracted from peripheral venous blood either by standard salt extraction or with a Nucleon kit (Gen-Probe Life Sciences).

**Figure 2:**
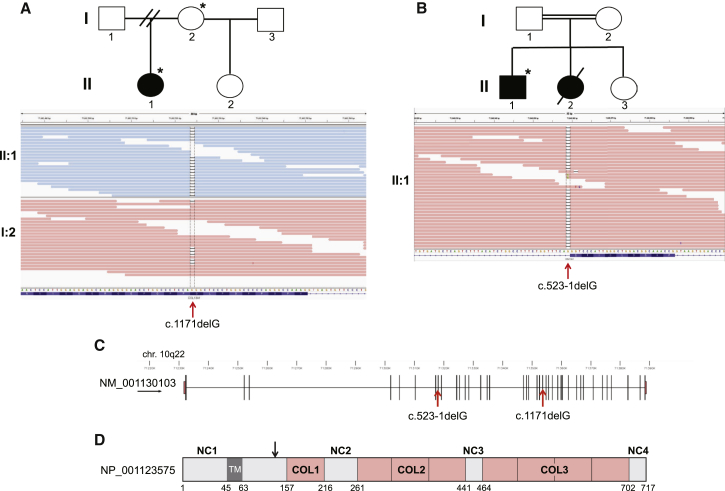
Sequence Analysis of *COL13A1* Mutations and Schematic Representations of *COL13A1* and COL13A1 (A) Top: pedigree of affected individual II:1 in family 1. Bottom: IGV pileup of sequencing reads for individual II:1 (blue) and her unaffected mother, I:2 (pink). The red arrow indicates the homozygous frameshift mutation c.1171delG. The mother is heterozygous for this mutation. Numbers at the top represent physical locations (based on Ensembl genome assembly GRCh37), and nucleotides and amino acid residues are shown below. Individuals analyzed by WES are indicated by asterisks. (B) Top: pedigree of affected individuals II:1 and II:2 in family 2. Bottom: IGV pileup of sequencing reads for individual II:1 (pink). The red arrow indicates the homozygous splice-site mutation c.523−1delG. (C) Schematic of *COL13A1*, encoding transcript variant 1 (GenBank: NM_001130103.1), in chromosomal region 10q22. Vertical black bars represent coding exons, and red arrows indicate the locations of pathogenic mutations. Numbers represent physical locations (Ensembl genome assembly GRCh37). (D) Schematic of the full-length COL13A1 isoform 1 (GenBank: NP_001123575), including a short intracellular domain, a single transmembrane helix (TM; dark gray), and the extracellular region with three collagenous domains (COL1–COL3) separated by short non-collagenous domains (NC1–NC4). Each collagen domain has separate collagen helix repeats (pink boxes). The approximate location of the proprotease recognition site is indicated by the black arrow. Numbers indicate the amino acid residues composing each domain.

WES was performed with Agilent SureSelect V5 Human All Exon baits (for individuals I:2 and II:1 in family 1; Figure 2A) or Roche NimbleGen SeqCap EZ Human Exome Library v.2.0 (for individual II:1 in family 2) and subsequent 100 bp paired-end sequencing on the Illumina HiSeq platform. Whole-genome alignment of FASTQ files was performed as described previously.^6^ We processed alignments in the SAM and BAM formats with SAMtools,^7^ Picard, and the Genome Analysis Toolkit (GATK).^8^, ^9^ Genomic VCFs were generated with the HaplotypeCaller function of GATK and subsequent joint calling of SNVs and indels with 217 other locally sequenced samples. Variants were recalibrated as per GATK guidelines,^10^ although no hard filtering was performed. The functional consequences of variants were determined with Ensembl’s Variant Effect Predictor.^11^ Using in-house-generated Perl scripts (see Web Resources), we removed variants that had a minor allele frequency of 1% or higher in dbSNP, the NHLBI Exome Sequencing Project (ESP) Exome Variant Server (VCF downloaded May 2014), the Exome Aggregation Consortium (ExAC) Browser (VCF downloaded November 2014), and locally sequenced exomes.

Because both families contain individuals without a molecular diagnosis of a CMS, we performed a combined analysis for affected individuals 1 (II:1 in family 1) and 2 (II:1 in family 2) in order to determine whether they share a genetic cause of their disease. We processed the variants that remained after filtering to identify either possible compound-heterozygous or possible homozygous “functional” variants (non-synonymous, splice consensus, or coding indel variants) with a Combined Annotation Dependent Depletion (CADD) Phred-like score ≥ 10. After this filtering step, only one candidate gene, *COL13A1*, remained with a biallelic pathogenic variant in both families (Table S1). For individual 1 (II:1 in family 1), we identified a homozygous frameshift variant (c.1171delG [p.Leu392Sfs^∗^71] [GenBank: NM_001130103.1]) in *COL13A1* (Figure 2A). For individual 2, we identified a homozygous splice-site mutation, c.523−1delG, in *COL13A1* (Figure 2B). This variant is predicted to allow splicing but lead to premature termination due to a single-base deletion in the coding sequence (p.Gly175Vfs^∗^20). Neither individual carries putative “functional” pathogenic variants in other known CMS-related genes. Sanger sequencing confirmed that individual 3 is also homozygous for the c.523−1delG splice-site mutation. Both mutations segregate with the disease in their respective families (Figure S1) and are absent in the 61,486 individuals of the ExAC dataset and 3,100 ethnically matched individuals in an in-house dataset. A further 12 individuals with a genetically undiagnosed but clinically secure CMS were screened by Sanger sequencing for mutations in *COL13A1*, but no further putative pathogenic variants were identified.

*COL13A1* is a large gene (it is 157 kb in size and has at least 38 exons; Figure 2C) that is ubiquitously expressed at low levels in many tissues, including connective tissues.^12^, ^13^, ^14^ It encodes the alpha chain of an atypical, non-fibrillar transmembrane collagen. COL13A1 (collagen type XIII alpha1 chain) consists of a short intracellular domain, a single transmembrane domain that anchors it to the plasma membrane, and a large, collagenous ectodomain (Figure 2D). The extracellular ectodomain contains three collagenous domains (COL1–COL3) separated by short non-collagenous domains (NC1–NC4) and a proprotease recognition site (Figure 2D), suggesting that the ectodomain can be proteolytically cleaved and shed into the extracellular matrix. COL13A1 forms the alpha chain of collagen XIII, which is trimeric like other collagens, and is thought to form homotrimers.^14^, ^15^ Collagen XIII interacts with fibronectin, heparin, and the basement-membrane proteins nidogen-2 and perlecan,^15^ indicating that it mediates multiple interactions with the extracellular matrix. COL13A1 has been previously implicated in regulating the maturation of the NMJ^16^ in either mouse models lacking the transmembrane anchoring domain^17^ or *Col13a1*^−/−^ animals.^16^ Although COL13A1 is present in the postsynaptic membrane and synaptic basement membrane and has been postulated as a potential CMS-associated protein,^18^ its precise molecular function is unknown.^16^

**Figure 3:**
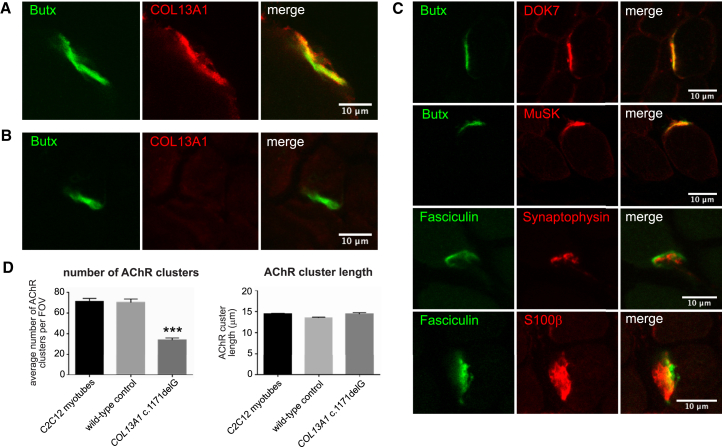
COL13A1 Is Localized at the Human NMJ and Mediates Clustering of AChRs (A) In human muscle, COL13A1 (red) is enriched at endplate regions of NMJs marked by α-bungarotoxin (Butx; green). Scale bar represents 10 μm. (B) Immunofluorescence labeling of quadriceps muscle from individual 1 (affected individual II:1 in family 1), who carries the homozygous frameshift mutation *COL13A1* c.1171delG, which causes loss of COL13A1 accumulation (red) at an NMJ marked by α-bungarotoxin (Butx; green). Scale bar represents 10 μm. (C) Normal expression and localization of markers for the NMJ (α-bungarotoxin [Butx]), the presynaptic terminal in NMJs (synaptophysin), terminal Schwann cells (S100β), the intersynaptic space (fasciculin), and postsynaptic proteins (DOK7 and MuSK) in individual 1. Scale bars represent 10 μm. (D) AChR-cluster analysis of the *COL13A1* c.1171delG variant. Bar graphs show that the frameshift mutation caused the number of AChR clusters in differentiated mutant myotubes to be statistically significantly lower than that in mock transfected (wild-type) or non-transfected C2C12 myotubes (left panel), but there was no significant effect on average cluster length (right panel). Statistical analyses used one-way ANOVA with Tukey’s multiple-comparison test (^∗∗∗^p < 0.001).

To further understand the localization and potential role of COL13A1 at the NMJ, we used the quadriceps muscle biopsy from individual 1 and human laryngeal muscles from unaffected individuals undergoing therapeutic laryngectomy as controls. Laryngeal muscles have a relatively small size and high endplate density, which facilitates visualization of stained endplates. Longitudinal and transverse 8–10 μm sections from fresh-frozen tissues were processed for immunofluorescence microscopy via standard methods. Images were captured on an IX71 Olympus microscope with Simple PCI software (Digital Pixel Imaging Systems) and analyzed with Volocity (PerkinElmer) or captured on a Zeiss LSM 510 inverted confocal microscope. We used antibodies against synaptophysin (SV2, Developmental Studies Hybridoma Bank, University of Iowa) to label the presynaptic terminal in NMJs or against S100 calcium-binding protein B (S100β, antibody SAB1402349, Sigma-Aldrich) to label terminal Schwann cells. We used Alexa Fluor 488-conjugated fasciculin (Life Technologies) or Alexa Fluor 488-conjugated α-bungarotoxin and antibodies against DOK7 (H-284, Santa Cruz Biotech) and MuSK (ab92950, Abcam) to label the intersynaptic space and key postsynaptic proteins. We used two different antibodies against the COL13A1 C-terminal region: affinity-purified guinea pig anti-COL13A1 antibody (Eurogentec), against amino acids 442–463 for full-length isoform 1 (GenBank: NP_001123575), or a rabbit polyclonal antibody (STJ92376, St. John’s Laboratory). The specificity of the Eurogentec and St. John’s Laboraroty antibodies was verified by immunocytochemical detection in HEK293 cells overexpressing full-length COL13A1 isoform 1 (Figure S2A) and by Western blots (Figure S2B), respectively. We then demonstrated that COL13A1 localized at the motor endplates in normal control muscle (Figure 3A) but that COL13A1 was absent in motor endplates from individual 1 (affected individual II:1 in family 1), consistent with the effect of the homozygous frameshift *COL13A1* mutation (c.1171del [p.Leu392Sfs^∗^71]) carried by this person (Figure 3B). However, other key NMJ proteins had normal localization at the motor endplates from individual 1 (Figure 3C). Western blots on protein extracts from human muscle, HEK293 cells, and HEK293 cells overexpressing COL13A1 isoform 1 (GenBank: NP_001123575) as a positive control demonstrated that endogenous COL31A1 was present in muscle (Figure S2B), consistent with previous reports.^16^ However, the endogenous protein in muscle had a lower apparent mass than did full-length COL13A1 isoform 1. RT-PCR confirmed that the endogenous muscle protein was produced from *COL13A1* transcript variant 21 (GenBank: NM_080798), encoding a smaller protein isoform (GenBank: NP_542988) (data not shown). Compared to the major transcript variant 1 (GenBank: NM_001130103.1), this transcript variant has exons 3, 5, 6 and 30 spliced out.

To determine the effect of COL13A1 loss on endplate development and NMJ maturation, we engineered the homologous *COL13A1* c.1171delG frameshift mutation into the immortalized mouse myoblast C2C12 cell line by using the CRISPR-Cas9 nickase genome-editing technology according to standard methods.^19^, ^20^ In brief, guide oligonucleotides and a repair-template oligonucleotide (Integrated DNA Technologies) were cloned into an adapted plasmid based on pX335-U6-Chimeric_BB-CBh-hSpCas9n(D10A),^19^ a kind gift from Feng Zhang (Addgene plasmid no. 42335). Guide A oligonucleotide sequences were 5′-CACCGAGAGATATGGAGCCCCAAAG-3′ (forward) and 5′-AAACCTTTGGGGCTCCATATCTCTC-3′ (reverse), and those for guide B were 5′-CACCGTGGCAGCAAAAACTCACCTT-3′ (forward) and 5′-AAACAAGGTGAGTTTTTGCTGCCAC-3′ (reverse). The repair template was 5′-CAGGGAGAAAAAGGT GATGCTGGCAATGCCATCGGAGGAGGCAGGGGGGAGCCTGGCCCCCCGGGGTCCCTGGGCCCCCTGGGCCAAAGGTGAGTTTTTGCTGCCATCTGCATTGAGAGAGATATGGAGCCCCAAAGAGTCACAG-3′. C2C12 cells were electroporated with 20 μg of plasmid and 10 μl repair-template oligonucleotide (10 μM) with a Neon electroporator (Life Technologies), selected with 1 mg/ml G418 (Life Technologies), and cloned by serial dilution in 96-well flat-bottomed culture plates. We assessed clones by PCR of genomic DNA by using the primer pair 5′-GGTTGACCCAGAAACCCCAA-3′ and 5′-ACTTATGACTCCATGCCCAGG-3′. Successful mutagenesis was confirmed by the creation of a new KpnI restriction site and the presence of the deletion after Sanger sequencing.

We then assessed the functional effect of the *COL13A1* c.1171delG mutation on AChR clustering after differentiation and fusion of cells into myotubes and the induction of AChR clusters by the addition of soluble neural agrin. AChR clusters were visualized with Alexa Fluor 594-conjugated α-bungarotoxin. The c.1171delG mutation caused the number of AChR clusters longer than 3 μm in differentiated myotubes to be approximately 50% lower than that in mock transfected (wild-type) or non-transfected C2C12 myotubes (Figure 3D). The average size of the AChR clusters was not affected (Figure 3D). Effects on postsynaptic maturation in vivo could come from either muscle-membrane-located COL13A1 or the proteolytically cleaved ectodomain of COL13A1. The effect on AChR clustering, and by implication on the formation and maintenance of NMJs, appears to be independent of any dramatic effect in vivo on key AChR-clustering-pathway proteins MuSK and DOK7 (Figure 3C).

Collagens are known to have important roles at the NMJ.^18^ Mutations in *COLQ* result in an endplate acetylcholinesterase deficiency,^21^ and collagen IV is thought to be involved in maintaining the integrity of motor nerve terminals.^22^ Previous studies of either mouse models lacking the transmembrane anchoring domain^17^ of Col13a1 or *Col13a1*^−/−^ animals^16^ have suggested that COL13A1 has an essential role in the organization of terminal Schwann cells and NMJ formation. Mouse models lacking the transmembrane anchoring domain exhibit rough and uneven myofibers, show vacuolization and enlargement of mitochondria, and are susceptible to exercise-induced muscle damage.^17^ We have not seen any clear evidence of a concomitant myopathy in individuals with *COL13A1* mutations, given that they all have normal CK levels, normal fiber-type distributions, and few central nuclei. However, H&E staining of muscle from individual 1 revealed peripheral vacuole-like areas (data not shown), and although an artifactual effect cannot be ruled out, this is consistent with observations in knockout animals. *Col13a1*^−/−^ animals grew more slowly than wild-type and heterozygous littermates, such that their general condition deteriorated with age. Immunocytochemistry studies showed defective maturation of the postsynaptic structures, which is consistent with our in vitro results (Figure 3D). Furthermore, some synaptic sites showed presynaptic defects, which included incomplete clustering of synaptic vesicles at nerve terminals. Importantly, nerve terminals did not always precisely oppose the postsynaptic AChR clusters, had projections into the synaptic cleft, and were often erroneously enwrapped by Schwann cells beyond the postsynaptic specializations. This would cause a decreased contact surface for neurotransmission. Furthermore, electrophysiological recordings from the *Col13a1*^−/−^ mice indicated impaired presynaptic function.^16^ By extrapolation from our findings and from the studies of mouse models, it is likely that the loss of COL13A1 from the synaptic cleft in these individuals affects both pre- and postsynaptic structural organization and thus function at the NMJ. A potential interacting partner on the presynaptic nerve terminal is the intergrin α1 subunit, which acts as a receptor for collagen XIII in cultured cells.^23^

In summary, we report human mutations in *COL13A1*. Our findings expand the genetic heterogeneity of CMSs and delineate an unusual form of this condition (CMS type 19). The phenotypes of the three affected individuals in this report most closely resemble CMS due to *RAPSN* mutations given that onset was at birth and included respiratory and feeding difficulties, slight dysmorphic facial features, and ptosis but normal eye movements. However, they differ from RAPSN-type CMS because of the presence of pectus carinatum, limited fatigability of the ptosis, and no beneficial response to anticholinesterase medication. Accurate molecular testing is essential for the differential diagnosis of CMS sub-types and other neuromuscular conditions because it can refine treatment and prognosis for individuals affected by these conditions. We note that characterization of the CMS for individual 1 prompted treatment with 3,4-DAP, which blocks voltage-gated potassium channels, and salbutamol, a β2-adrenergic receptor agonist, which had good clinical effect. The β2-adrenergic receptor agonists salbutamol and ephedrine have been found to be beneficial for CMS forms (such as CMS type 10) caused by *DOK7* mutations that affect the maturation and maintenance of NMJ structure, whereas 3,4-DAP enhances acetylcholine release from the presynaptic nerve terminal. The beneficial effects of salbutamol would be consistent with the fact that *COL13A1* mutations affect the maturation and maintenance of the synaptic structure, and the molecular characterization of this condition has facilitated the implementation of appropriate treatment strategies. Our results emphasize the crucial role of extracellular-matrix proteins, other than those in the agrin pathway, in the formation and maintenance of the synapse and highlight the importance of collagen XIII in NMJ cytoarchitecture and neurotransmission.

## Figures and Tables

**Table 1:** Clinical Features of Individuals with Mutations in *COL13A1*

		**Family 1**	**Family 2**	
**II:1**	**II:1**	**II:2**
Consanguinity		no	yes	yes
Mutation (type)		c.1171delG (frameshift)	c.523−1delG (splice-site or frameshift)	c.523−1delG (splice-site or frameshift)
Sex		female	male	female
Current age		24 months	27 years	died at 8 years
Age at assessment		5 months	24 years	5 years
Age of onset		birth	birth to 1 year	birth
Manifesting symptoms		BD, FD	ptosis, FD	BD, FD
Ptosis		+	+	+
Ophthalmoparesis		−	−	−left, +right
Facial weakness		−	+	+
Bulbar weakness		+	−	+
Proximal weakness	upper limbs	+	−	+
lower limbs	+	−	+
Distal weakness	upper limbs	+	+	+
lower limbs	+	−	+
Axial weakness		+	−left, +right	+
Distal-joint laxity		+	−	+
Contractures		−	−	−
Spinal rigidity		−	+	+
Dysmorphic features		+	+	+
Mild learning difficulties		NK	+	NK
Decrement on RNS		+	+	NA
Abnormal jitter		+	+	NA
Other		LRTI	LRTI	CLD, hh, LRTI
Response to treatments		DAP, sb, NIV, py-ve	none	NIV, py-ve

Abbreviations are as follows: BD, breathing difficulty; CLD, chronic lung disease; DAP, 3,4-diaminopyridine; FD, feeding difficulty; hh, hiatus and diaphragmatic hernia; LRTI, lower-respiratory-tract infection; NA, not assessed; NIV, non-invasive ventilation; NK, not known; py-ve, no response to pyridostigmine treatment; RNS, repetitive nerve stimulation; and sb, salbutamol.

## Web Resources

The URLs for data presented herein are as follows:

Ensembl genome assembly GRCh37, http://grch37.ensembl.org/Homo_sapiens/
Exome Aggregation Consortium (ExAC) Browser, http://exac.broadinstitute.org
NHLBI Exome Sequencing Project (ESP) Exome Variant Server, http://evs.gs.washington.edu/EVS/
OMIM, http://www.omim.org
Perl scripts, https://github.com/gantzgraf/vcfhacks
RefSeq, http://www.ncbi.nlm.nih.gov/refseq/

## Supplemental Data

Document S1. Supplemental Note, Figures S1 and S2, and Table S1

Document S2. Article plus Supplemental Data
