# Supplementary material for: Congenital Myasthenic Syndrome Type 19 Is Caused by Mutations in COL13A1, Encoding the Atypical Non-fibrillar Collagen Type XIII α1 Chain
Source: Am J Hum Genet. 2015 Nov 25;97(6):878–85. doi: 10.1016/j.ajhg.2015.10.017 (PMC4678414; doi:10.1016/j.ajhg.2015.10.017)
Supplement: Document S1. Supplemental Note, Figures S1 and S2, and Table S1 [file mmc1.pdf]

The American Journal of Human Genetics
Supplemental Data

Congenital Myasthenic Syndrome Type 19 Is Caused by Mutations in *COL13A1*, Encoding the Atypical Non-fibrillar Collagen Type XIII α1 Chain

Clare V. Logan, Judith Cossins, Pedro M. Rodríguez Cruz, David A. Parry, Susan Maxwell, Pilar Martínez-Martínez, Joey Riepsaame, Zakia A. Abdelhamed, Alice V.R. Lake, Maria Moran, Stephanie Robb, Gabriel Chow, Caroline Sewry, Philip M. Hopkins, Eamonn Sheridan, Sandeep Jayawant, Jacqueline Palace, Colin A. Johnson, and David Beeson

Supplemental Note: Case Reports

Individual 1 (affected individual II:1 in family 1; **Table 1**) of white European origin was born after a normal pregnancy. Soon after birth, she required intermittent ventilation for apneas. General hypotonia and poor suck were also noted, and therefore she was fed via nasal-gastric tube. At 7 months a Nissen fundoplication was performed to treat gastroesophageal reflux and a gastrostomy-feeding device fitted. During infancy she had recurrent episodes of sudden deterioration in respiratory function lasting days or weeks related to respiratory infections, needing non-invasive ventilation. Examination showed mild bilateral non-fatigable ptosis with normal eye movements, no facial weakness, but poor head control and marked neck weakness as well as limb hypotonia, with inability to sit. There were no joint contractures. By the age of two cognitive developmental delay was evident and dysmorphic features were noted in the face and chest, with low-set ears, micrognathia, retrognathia, high-arched palate, and *pectus carinatum*. A muscle biopsy from the quadriceps muscle at 6 months of age revealed abnormal variation in fibre size, with several populations of fibres staining positive for foetal myosin (**Figure 1A-D**). Respiratory enzymes were normal, with many mitochondria appearing swollen (**Figure 1A-D**). Whole-muscle MRI was normal. Serum creatine kinase levels were within normal range. Stimulated single fibre electromyography (SFEMG) for the left *orbicularis oculi* muscle showed grossly increased jitter values and many blocks (mean MCD of 133.36 ±41.9µs and 14% blocking), consistent with a diagnosis of CMS. Repetitive nerve stimulation in right *abductor digiti minimi* and left *flexor hallucis brevis* showed significant decrement >20% at rest to low stimulation rates (**Figure 1E**). Treatment with pyridostigmine (1mg/kg/day) did not have a significant effect on muscle strength. Subsequent treatment with 3,4-diaminopyridine (0.3 mg/kg/day) and salbutamol (0.56 mg/kg/day) produced a remarkable improvement in her motor function and respiratory function: head control improved, and she could achieve unassisted sitting. Non-invasive ventilation requirement went down to nocturnal only and episodes of respiratory deterioration abated.

Individuals 2 and 3 (affected individuals II:1 and II:2 in family 2; **Table 1**) are siblings with parental consanguinity whose family came from the Indian subcontinent. Individual 2 (individual II:1) was born after a normal pregnancy. He required ventilation for the first few days after birth and subsequently had feeding difficulties in the first year of life. He was noted to have ptosis shortly after birth. Although his motor milestones were normal, during childhood he had dyspnoea on exertion, recurrent chest infections and mild learning difficulties. The course of disease was stable, with lack of progression and even improvement over the years. At the age of 25, examination showed constant moderate bilateral ptosis (30%), mild weakness of eye closure and facial movements, and mild weakness of hip extensors. There was facial dysmorphism, with micrognatia, low-set ears, and a high arched palate (**Table 1**), in addition to skeletal abnormalities such as *pectus carinatum* and marked

bilateral *pes cavus*. There was a degree of spine rigidity with mild limitation of neck extension but no distal contractures. Neurophysiology was performed at age 18. Repetitive nerve stimulation at 3Hz in the right *anconeous* muscle showed significant decrement >20%. SFEMG in right *extensor digitorum communis* showed 50% increased jitter with 0% blocking and mean MCD of 69.8±34.4µs. Treatment with pyridostigmine up to 6 mg/kg/day had no effect.

Individual 3 (affected individual II:2 in family 2; **Table 1**) was more severely affected than her brother. She was born after a normal pregnancy, and had severe breathing and feeding difficulties after birth, partly due to a combined hiatus and diaphragmatic hernia. She was oxygen-dependent until age 2 and had recurrent chest infections, after which she developed chronic lung disease. She had delayed motor milestones and did not walk until age 2. She remained predominantly gastrostomy fed and had limited walking and exercise tolerance with progressive fatigue. Examination at age 5 years showed bilateral ptosis, mild limitation of eye movements and mild facial weakness. Muscle bulk was reduced generally, although all muscles other than neck flexors had only mild weakness. However, she had difficulty jumping and was unable to hop. Facial dysmorphism was similar to her brother, Individual 2. In addition *pectus carinatum* and a degree of spinal rigidity with kyphotic posture, predominantly at the cervical-thoracic region, were present but no distal contractures. She had a Tensilon test with no obvious improvement. Pyridostigmine was not initiated due to parents' choice. This individual died at age 8 due to severe respiratory problems related to muscle weakness and her long-standing chronic lung disease.

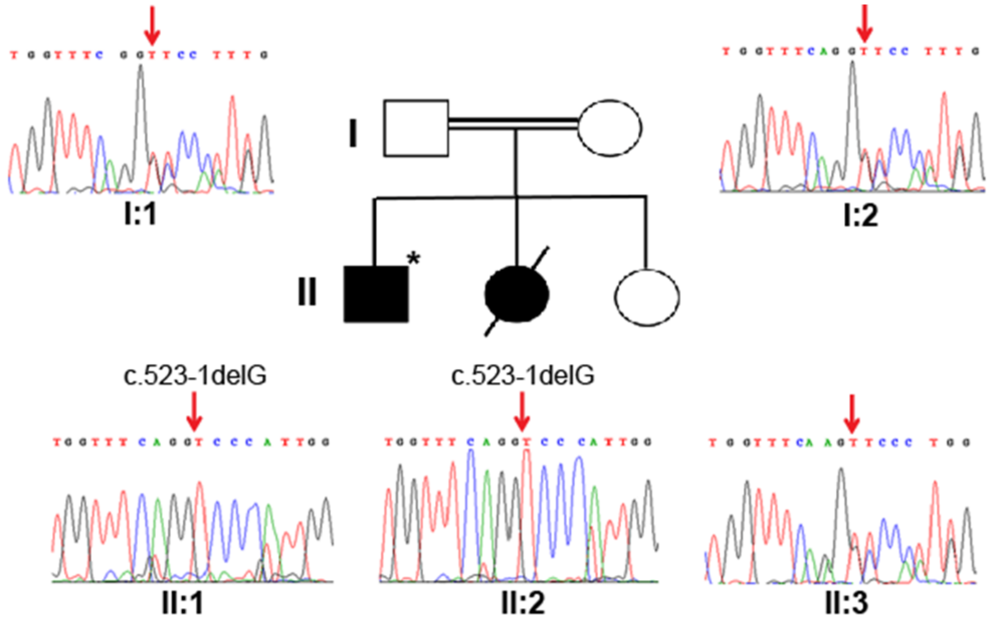

Figure S1. **Segregation of *COL13A1* c.523-1del splice-site mutation in family 2**

Electropherograms showing the segregation of the *COL13A1* c.523-1del splice-site mutation with disease in the pedigree of family 2. Both affected siblings, II:1 and II:2, are homozygous whereas the unaffected sibling II:3 and the parents (I:1 and I:2) are heterozygous carriers (red arrows)

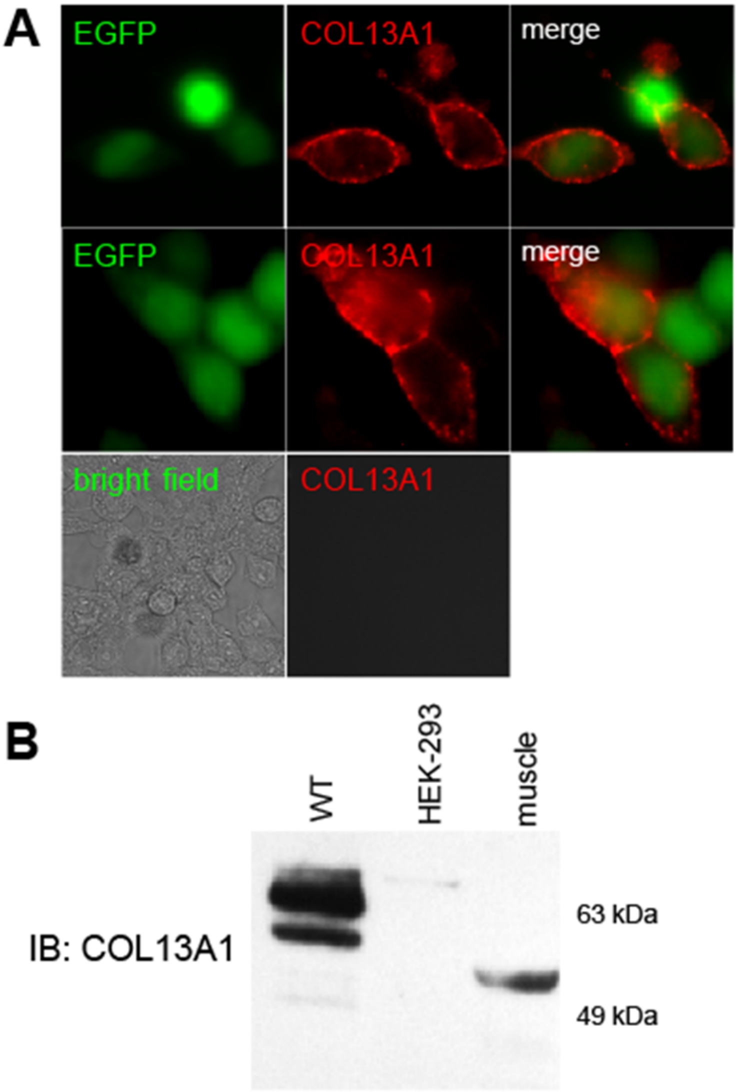

Figure S2. **Validation of commercial antibody specificities against the COL13A1 C-terminus**

(A) Specific recognition of over-expressed COL13A1 at the cell surface of HEK293 cells transfected with human full-length COL13A1. We purchased an IMAGE clone containing cDNA from transcript variant 1 (RefSeq NM_001130103.1) from Source BioScience Ltd., Nottingham, UK (clone number 9020372), subcloned into a mammalian expression vector (pcDNA3.1-hygro (-), Life Technologies Ltd., Paisley, UK), and verified by Sanger sequencing. HEK293 cells were co-transfected with the wild-type *COL13A1* cDNA and an eGFP construct, plated on coverslips coated with poly-L-lysine (Sigma-Aldrich Co. Ltd., Gillingham, UK) and visualized using standard immunocytochemical staining techniques 48 h after transfection. Cells were incubated for 1 hour with affinity-purified guinea pig anti-COL13A1 antibody against the C-terminal region (Eurogentec SA, Liege Science Park, Belgium) diluted in DMEM, 1% bovine serum albumin (BSA) and 20 mM HEPES. Cells were rinsed with PBS, fixed with 3% paraformaldehyde and then labelled with Alexa Fluor-568 goat anti-guinea pig IgG (H+L) (Life

Technologies Ltd., Paisley, UK) at x750. Coverslips were mounted on slides using Confocal-Matrix (Micro-Tech-Lab , Graz, Austria). Images were captured on an IX71 Olympus microscope using Simple PCI software (Digital Pixel Imaging Systems, Brighton, UK).

(B) Western blot analysis of COL13A1-transfected HEK-293 cells (WT), untransfected cells as negative control (HEK-293) and human muscle tissue. Lysates were prepared from cells or human muscle using standard methods. Total soluble protein of 25 µg was separated by SDS-PAGE and over-expressed or endogenous COL13A1 isoforms were immunodetected with rabbit anti-COL13A1 C-terminal region (St. John's Laboratory Ltd., London UK). Bands of different molecular mass correspond to different splice variants of transcripts. Over-expressed full-length COL13A1 isoform 1 (RefSeq NP_001123575) has length 717aa and predicted mass 70.0 kDa, whereas the endogenous muscle protein is produced from transcript variant NM_080798 (see main text for details) corresponding to protein isoform NP_542988 (length 645aa, predicted mass 63.0 kDa).

Table S1. Variant filtering strategy for the analysis of whole exome sequencing data

	**Individual 1**	**Individual 2**	**Individuals 1 & 2**
Genes with functional, potentially biallelic variation	4071	3184	2318
… minus 'common' variants from 217 local exomes	187	174	9
… minus variants with MAF ≥ 1%	33	29	1
… minus variants with a CADD PHRED-like score < 10	18	15	1

This combined the analysis of variants for individual 1 (affected individual II:1 in family 1) with variants identified for individual 2 (affected individual II:1 in family 2). The table lists the number of genes with functional biallelic variants called by Haplotype Caller predicted to affect either coding or splicing that remained after each filtering step.
